# Rats move nesting materials to create different functional areas: Short report

**DOI:** 10.1177/00236772221122132

**Published:** 2022-09-29

**Authors:** Lucia Amendola, Nanqi Xu, Daniel M Weary

**Affiliations:** The University of British Columbia, Vancouver, Canada

**Keywords:** Environmental enrichment, cage cleaning, animal welfare

## Abstract

Here we document how rats separate their living space into different functional regions. Five groups of four female Sprague Dawley rats were housed in caging systems that consisted of two standard cages connected by a tube. Both cages were provided with the same amount of bedding and nesting materials, but only one contained food and water. Nesting cover and weight of each cage were measured once a week for five weeks during cage cleaning. We found that the cages with food and water had 9% less nesting material coverage but had gained 90% more weight when compared with cages where food and water were absent. These results indicate that, when provided with separate spaces, rats move nesting materials away from the cage containing food and water sources, but preferentially excrete in the cage with water and food.

## Les rats déplacent les matériaux de nidification pour créer différentes zones fonctionnelles Résumé

Nous avons ici documenté comment les rats séparent leur espace de vie en différentes régions fonctionnelles. Cinq groupes de quatre rats Sprague Dawley femelles ont été logés dans des systèmes de mise en cage composés de deux cages standard reliées par un tube. Les deux cages ont reçu la même quantité de litière et de matériel de nidification, mais une seule contenait de la nourriture et de l'eau. La couverture de nidification et le poids de chaque cage ont été mesurés une fois par semaine pendant cinq semaines pendant le nettoyage de la cage. Nous avons constaté que les cages contenant de la nourriture et de l’eau avaient 9% de matériel de nidification en moins, mais qu’elles avaient gagné 90% de poids en plus par rapport aux cages ne contenant ni nourriture ni eau. Ces résultats indiquent que, lorsqu’ils disposent d’espaces séparés, les rats éloignent les matériaux de nidification de la cage contenant des sources d’eau et de nourriture, mais qu’ils excrétent préférentiellement dans la cage contenant de l’eau et de la nourriture.

## Ratten verlagern Nistmaterial zur Schaffung verschiedener Funktionsbereiche Abstract

Hier haben wir dokumentiert, wie Ratten ihren Lebensraum in verschiedene funktionale Bereiche aufteilen. Fünf Gruppen mit vier weiblichen Sprague-Dawley-Ratten wurden in Käfigsystemen untergebracht, die aus zwei durch ein Rohr verbundenen Standardkäfigen bestanden. Beide Käfige waren mit der gleichen Menge an Einstreu und Nistmaterial ausgestattet, aber nur einer enthielt Futter und Wasser. Das Nistmaterial und das Gewicht jedes Käfigs wurden fünf Wochen lang einmal pro Woche während der Käfigreinigung gemessen. Wir stellten fest, dass die Käfige mit Futter und Wasser 9% weniger Nistmaterial enthielten, aber 90% schwerer waren als die Käfige ohne Futter und Wasser. Diese Ergebnisse legen nahe, dass Ratten, wenn ihnen getrennte Räume zur Verfügung stehen, Nistmaterial von dem Käfig mit Futter- und Wasserquellen wegbringen, aber vorzugsweise im Käfig mit Wasser und Futter ausscheiden.

## Las ratas mueven materiales de nidos para crear diferentes áreas funcionales Resumen

Aquí documentamos cómo las ratas separan su espacio vital en diferentes secciones funcionales. Cinco grupos de cuatro ratas Sprague Dawley hembras fueron enjaulados en dos jaulas estándar conectadas por un tubo. Ambas jaulas estaban provistas de la misma cantidad materiales para anidar, pero solo una contenía comida y agua. Se midió la cobertura de los nidos y el peso de cada jaula una vez a la semana durante cinco semanas durante la limpieza de las jaulas. Descubrimos que las jaulas con comida y agua tenían un 9% menos de cobertura de material de nidos pero habían ganado un 90% más de peso en comparación con las jaulas en las que no había comida ni agua. Estos resultados indican que, cuando se les proporcionan espacios separados, las ratas alejan los materiales para hacer nidos de la jaula que contiene las fuentes de agua y comida, pero excretan preferentemente en la jaula con agua y comida.

In the wild, rats divide their living spaces into different functional regions, including sites for nesting, food storage or hoarding, mating, and excreting,^1,2^ but housing systems for laboratory rodents provide limited opportunities for spatial segregation of these behaviors.^3^ Several studies from our research group have used two-cage systems (two standard cages connected by a tube) to house rats.^4,5^ Our informal observations suggested that rats sometimes divide their living space into different functional areas; for example, showing preferential use of one of the cages to sleep. The aim of this study was to document the differential use of cages when rats were housed in a two-cage system.

**Figure 1.**
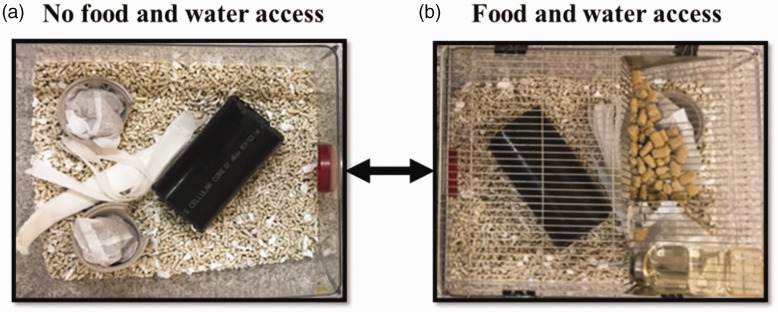
The two-caging systems showing that cages ((a) and (b)) were provided with the same amount of nesting materials and (b) Cage, with the lid in place, illustrating that only one of the cages was provided food and water.

All housing and procedures followed the guidelines on the care and use of rodents in research established by the Canadian Council on Animal Care (CCAC) and were approved by the Animal Care Committee (protocol A15-0071) of The University of British Columbia (a CCAC certified institution). We used a convenience sample of five groups (four rats per group) of nine-month-old female Sprague-Dawley rats that were made available to us as surplus animals; no a priori power analysis was performed. Each group was housed in a pair of standard cages (20 cm × 50 cm × 40 cm) connected by a red tinted polycarbonate tube (7.6 cm diameter, 15 cm long). Each cage contained around 1650 g of bedding material (Enrichment Bedding, Biofresh, Absorption Corp, WA, USA), 15 g of nesting materials (shredded paper towels, two cardboard cups and two bags of Bed-r Nest (The Andersons, Inc., Maumee, OH, USA)) and a PVC tube (Figure 1(a)). Cages were cleaned once a week and approximately 15 g of additional nesting materials were added to both cages four days after cage cleaning. Ad libitum food (Rat Diet PMI 5012, Lab Diets, Land O’Lakes, Inc., MN, USA) and tap water were provided in just one of the cages of the system (Figure 1(b)). Before starting the study, food and water were always placed in the right-side cage of the system. During the study the position of food and water was alternated weekly between cages, starting one week before data collection started.

Photos of each cage were taken immediately before cage cleaning using a camera positioned 25 cm above the cage (Supplementary material, Images, online). Photographs, blinded for cage identity and food and water location, were analyzed using Image J (Schneider et al., 2012)^6^, estimating the percentage of coverage of each element (bedding, feces and nesting materials, such as carboard cups, Bed-r Nest and paper towels) with the Analyze Particles function (Supplementary material, Methods). The results of this method were consistent with those from visual scoring by a trained observer (also blind to group and food and water location; see Supplementary material, Methods). Bedding and nesting materials added to each cage prior to cage change were weighed. During cage change, the contents of each cage (excluding the PVC tube) were transferred into a plastic bag and weighed.

Percentage of nesting material coverage (i.e. cover of paper towels, carboard cups and Bed-r Nest) and weight change (g) over each week of use were averaged across the five weeks of observations providing a mean value for each of the five pairs of cages. The within-cage-pair difference in these measures was then assessed using paired *t*-tests, comparing cages with and without provision of food and water. Statistical analysis was carried out using R (R Development Core Team, Version 3.6.2) and RStudio (RStudio, Inc., Version 1.2.5033). Normality was assessed visually, and all results are presented as mean ± standard error.

**Figure 2.**
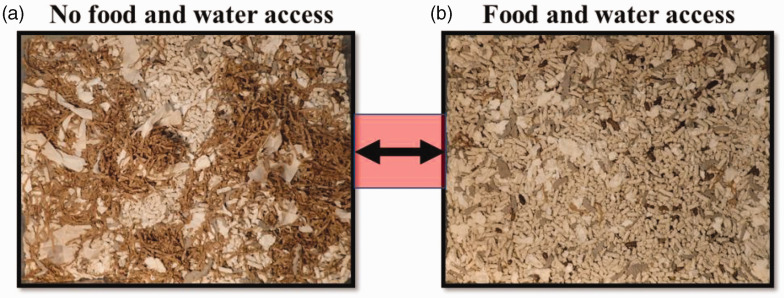
One example of a two-caging system after a week of use. (a) The cage that did not contain food and water and (b) the cage where food and water were provided (images are available via the UBC Faculty of Land and Food Systems dataverse: https://doi.org/10.5683/SP3/YLAXKG).

After a week of use cages with food and water had on average 9% less nesting material coverage than did cages without food and water (53.3 ± 0.7 *vs*. 58.7 ± 0.5% of cage floor coverage; *t* = –8.34, df = 4, *p* < 0.01; Figure 2). Both cages increased in weight over a week of use, but this increase was almost twice as high in cages containing food and water (356 ± 47 *vs*. 188 ± 14 g; *t* = 3.96, df = 4, *p* < 0.05).

These results suggest that rats segregated their living space by moving nesting materials between cages. This finding is consistent with results for mice housed in multi-cage systems; mice moved nesting materials away from the cage containing food and water.^3^ Both types of cages increased in weight, but this increase was greater for cages providing access to food and water. This difference may have been due in part to water flooding or food spillage, but excess food and water were never observed during cage cleaning. The cage with food and water appeared to contain more fecal boli, suggesting that rats (like mice^1–3^) preferred to use the cage with access to food and water as a latrine.

We conclude that, when provided the opportunity, rats segregate their living space into different functional areas. Two-cage systems are simple to implement and may represent a refinement over standard housing. Mice housed in the multi-caged systems show evidence of improved welfare. For example, mice housed this way perform higher frequencies of affiliative interactions and appear to be more resilient to the disturbance associated with cage cleaning than mice housed in standard cages.^3^ Future research is required to determine how generalizable these results are across multi-cage systems, to document rats moving nesting material and defecation and urination patterns, to assess the effects of multi-cage housing on social dynamics and nest quality, and to determine whether other welfare benefits are evident when rats are better able to segregate their living space in this way.
